# Intelligent Urban Traffic Congestion Prediction Through Accident-Aware and Time-Dependent Traffic Analytics

**DOI:** 10.3390/s26144629

**Published:** 2026-07-21

**Authors:** Akbar Ali, Noureen Zafar, Saleh Albahli, Muhammad Shiraz

**Affiliations:** 1 Department of Computer Science, Federal Urdu University of Arts, Science and Technology, Islamabad 44000, Pakistan; 2 University Institute of Information Technology, Pir Mehr Ali Shah Arid Agriculture University, Rawalpindi 46000, Pakistan; 3 Department of Information Technology, College of Computer, Qassim University, Buraydah 51452, Saudi Arabia

**Keywords:** traffic congestion prediction, road accidents, peak hours, fuzzy logic, ANPR sensors, GRU, Intelligent Transportation Systems

## Abstract

Rapid urban population growth has intensified traffic congestion in smart cities. This has resulted in longer travel times, higher fuel consumption, increased environmental pollution, greater operational costs, and slower emergency response services. Existing traffic congestion prediction models primarily rely on traffic-flow and temporal features; the effects of road accidents and peak-hour conditions are not adequately addressed. This limitation is particularly significant in smart cities where both recurrent congestion (peak-hour demand) and non-recurrent congestion (road accidents) influence traffic conditions they have a significant impact on the performance of the road network. This study introduces a novel Historical Accident-Aware Peak-Hour GAN-GRU (APG-GRU) framework. The proposed framework employs a data processing pipeline integrating traffic data with historical accident-related features to predict traffic congestion using these features. Extensive experiments are conducted on a novel integrated dataset consist on Automatic Number Plate Recognition (ANPR) traffic data and ANPR traffic observations with historical accident features. The results demonstrate that the APG-GRU framework achieved superior performance on the integrated features dataset, attaining an accuracy of 97.50%, a congested precision of 91.86%, a congested recall of 97.31%, and a congested F1-score of 94.51%, outperforming both the ANPR traffic-only dataset and all baseline models. The APG-GRU framework significantly outperforms a suite of benchmark models, including XGBoost, Long Short-Term Memory (LSTM), and Random Forest as baselines, which achieved accuracies between 84% and 95.5% with correspondingly lower precision, recall, and F1-scores. External validation using a traffic dataset collected from Lahore, Pakistan, further demonstrated the robustness and generalizability of the proposed APG-GRU framework. A web-based interface developed for the APG-GRU framework to visualize accident hotspots and route-level traffic conditions. Routes with smooth traffic flow are highlighted in green, whereas congested routes are highlighted in red, demonstrating the practical applicability of the proposed framework for smart city traffic management systems.

## 1. Introduction

One of the most apparent issues in urban transport is traffic congestion [1,2]. This leads to reduced road network efficiency, increased travel time, higher fuel consumption, and delays affecting public transport, private vehicles, logistics operations, and emergency services [3]. Congestion is created by recurring and non-recurring conditions that exist in many urban places developing today [4,5]. Typically, recurring congestion occurs at known and consistent peak-hour times, like in the morning or evening. Non-recurring congestion is when traffic congestion occurs due to unforeseen events like road accidents [6], vehicle breakdowns, road work, unusual weather, protests, or traffic enforcement efforts [7]. Such events disrupt the normal flow, and a prediction model trained to learn the regular traffic patterns may be blind to such events.

Road accidents are particularly significant for having an impact on both the safety of traffic and traffic flow [8,9]. Road traffic deaths still pose a major problem in global road safety, with 1.19 million deaths per year, as reported in the “Global status report on road safety 2023” (World Health Organization, 2023) [10]. When an accident happens, it can close one or more lanes, limit roadway capacity, cause bottlenecks, generate secondary conflicts, and prolong travel times for adjacent lanes [11]. According to [12] the type of accident, the number of vehicles involved, and the time of the accident have an impact on traffic accident-related congestion, suggesting that the context of the traffic accident should be taken into consideration when predicting traffic. Similarly, ref. [8] highlighted the importance of the propagation of congestion across road networks, wherein the states of traffic are influenced by dynamic spatiotemporal relationships [13].

The smart city system is an important opportunity to build data-driven traffic prediction as it is equipped with traffic surveillance and vehicle management infrastructure while having ANPR capability [14]. According to the official “Safe City” page of Islamabad, the infrastructure has cameras as part of the Intelligent Video Surveillance and Vehicle Management System (VMS) with ANPR facilities and is helpful for traffic regulation in the city [15]. The infrastructure produces a massive amount of spatiotemporal data suitable for intelligent transportation analysis. But raw ANPR data cannot always be used to accurately identify congestion, as congestion is not purely driven by the number of vehicles on the road. The traffic state may also change due to peak hours, recent accidents, traffic violations, and accident proximity.

The proposed APG-GRU framework takes ANPR features, peak-hour features, fuzzy logic-based congestion labels, accident-aware features, Generative Adversarial Network (GAN)-based balancing, and Gated Recurrent Unit (GRU)-based temporal classification. It also serves as a comparative model family for Random Forest, LSTM, XGBoost, and GRU. The key research hypothesis is that contributing a traffic congestion prediction framework with recurring factors like hour of day and/or peak-hour status along with non-recurring accident indicators increases the accuracy of the model’s prediction. Four scenarios representing different features are therefore compared to perceive whether the use of accident features, fuzzy logic-based labeling or data balanced using GAN improves the accuracy, congested precision, congested recall, and congested F1-score of the prediction. The most important class is the congested class as the aim of an intelligent traffic management system is to detect and respond to congestion before it turns into a severe condition.

### Research Contributions

The proposed APG-GRU framework advances our earlier work by moving the focus from weather-aware traffic congestion prediction toward historical accident-aware and peak-hour-based congestion prediction. While the previous F-GGRU framework mainly considered traffic, weather, and temporal features, the present study integrates smart city ANPR traffic observations with historical accident dataset features. This integration allows the framework to capture regular congestion patterns caused by peak-hour traffic demand as well as non-recurring congestion patterns associated with accident-related disturbances. Another important contribution of this work is the development of a web-based dashboard for the APG-GRU framework. The dashboard provides visual support for identifying accident hotspots and showing the selected route status as Smooth or Congested based on historical traffic and accident data. These additions distinguish the proposed APG-GRU framework from the previous F-GGRU framework and related traffic prediction studies by combining accident-aware feature integration, congestion prediction, and practical dashboard-based visualization within a single framework.

## 2. Related Work

### 2.1. Traffic Congestion Prediction in Smart Cities

The prediction of traffic congestion is a field of research that has received a great deal of attention in Intelligent Transportation Systems (ITS) as real-time traffic estimates are useful in routing, signal control, resource planning, emergency response, and urban policy. Previous techniques involved statistical models and classical machine learning, and due to the sequential and spatial nature of traffic data, deep learning (DL) has become more popular in recent years. In a research study, ref. [7] suggested a probabilistic congestion modeling using a Bayesian network approach and demonstrated that uncertainty-based approaches can be adopted to model congestion as a probability as opposed to a fixed class. This is relevant because congestion is a function of combinations of speed, flow, density, time, and other contextual features.

Deep learning models are widely used in traffic prediction due to their ability to capture nonlinear and temporal patterns. In a research study, ref. [16] suggested a hybrid feature space for smart city traffic prediction and found that road network features, weather, holidays, and peak hours are among the reasons for congestion of arterial roads; however, they do not address the issues relevant to accidental data. Their work also helps in the use of smart city traffic prediction using GRU and LSTM-based models that are able to learn temporal dependencies in the heterogeneous urban data. In recent years, ref. [17] introduced an F-GGRU framework which integrates Safe City traffic data, Open Weather data, peak-hour indicators, fuzzy labeling, GAN balancing, and GRU modeling for weather-aware congestion prediction. The present article builds on this trend and puts the focus on road accidents and peak-hour effects in the proposed framework.

### 2.2. Road Accidents and Non-Recurring Congestion

One of the most important limitations of various traffic congestion models is poor representation of non-recurring events. Congestion caused by an accident is not the same as congestion caused by a routine occurrence, as it can suddenly occur, causing congestion not only during the usual peak hours but also at other times of day or night. The traffic accidents that occur in cities are also important factors contributing to congestion in the urban road network, which is analyzed and presented in terms of accident type [12]. Accident vehicles and the time of occurrence are important factors affecting traffic accidents. This implies that accident data should be seen as context, as a predictor and not as post-event descriptive data.

A recent study also benefited from congestion modeling that is aware of accidents. The study [11] suggested a Bayesian Network for the analysis and prediction of congestion using accident data, and also proposed labeling strategies for congestion based on accident data. Their study presented that it is possible to model the causal effect of road accidents and congestion with accident data. Similarly, ref. [8] demonstrated the propagation of congestion using a dynamic Bayesian graph convolutional learning approach, which implies that accidents might also impact links and time windows around the incident location.

### 2.3. Fuzzy Logic for Congestion Labeling

Congestion is not always a well-defined state. Two vehicle speeds, 19 km/h and 21 km/h, can be very similar driving conditions, but be marked as different with the hard threshold [18,19]. Fuzzy logic is used to deal with this ambiguity by assigning membership degrees to linguistic categories like low, medium, and high speed. The study [19] proposed a two-stage fuzzy traffic congestion detector with actual motorway data and claimed that fuzzy methods are advantageous as drivers also understand the traffic condition qualitatively and not through crisp thresholds. The new idea introduced in the proposed framework is to use fuzzy labeling to create the congestion labels, which will be more flexible than only one fixed threshold.

### 2.4. GAN-Based Class Balancing

Traffic congestion datasets are typically imbalanced, meaning that the number of observations of non-congested traffic will be more common than congested traffic [20,21,22]. A small size of the minority class can lead to a high accuracy of a classifier for the majority class but low accuracy for the congestion class. To improve learning under rare or imbalanced conditions, GAN-based data generation has been used in traffic and mobility studies to generate realistic samples. The research study [23] applied a GAN-based method for online traffic-flow prediction with multi-source data, and ref. [24] proposed the Traffic GAN for network-scale traffic generation. Based on these studies, it can be concluded that generative models can be used as a data augmentation tool and also as a scene representation tool in the traffic system.

### 2.5. Machine Learning and Deep Learning Models for Comparative Evaluation

This article positions two machine learning models and two DL models in the comparative framework. Random Forest and XGBoost are chosen as machine learning baselines due to their proven effectiveness in handling nonlinear relationships between traffic features and also being robust tabular data models. LSTM and GRU are chosen as deep learning baselines due to their ability to process sequential data and their variants of recurrent neural network architecture. The reason for choosing GRU as the proposed model for the APG-GRU framework is that it is generally used with fewer parameters than LSTM in learning temporal dependencies, which is suitable for practical applications when it comes to the traffic prediction system, where a fast execution of the APG-GRU framework is required.

## 3. Dataset Construction and Feature Selection

The traffic and accident information used in this study are collected from two urban traffic environments to evaluate both the effectiveness and generalizability of the proposed APG-GRU framework. The primary experimental vehicular traffic and historical accident datasets are obtained from the Safe City Islamabad, Pakistan, surveillance system. The study area consists of selected major corridors, road segments, and urban sectors monitored through ANPR cameras. The APG-GRU framework uses synchronized vehicle traffic observations and historical accident records collected during March 2025 for the primary experimental evaluation. Although the available ANPR traffic dataset spans the period from January 2024 to December 2025, historical accident data recording began in March 2025. Since the proposed framework requires synchronized traffic and accident information, March 2025 is selected because it provides the required temporal overlap between ANPR traffic observations and accident records. This enabled the construction of an integrated accident-aware dataset in which traffic-flow, temporal, peak-hour, and accident-related features are aligned using date, time, camera location, geographic sector, and police-station information. The ANPR traffic data are collected from 25 monitoring cameras across Islamabad. A total of 869 accident records are initially retrieved from Safe City Islamabad, and after preprocessing, validation, duplicate removal, and incomplete-record filtering, 820 valid accident records are retained. The final cleaned and integrated Islamabad dataset contains 52,000 traffic observations incorporating traffic-flow features, speed-related features, peak-hour indicators, accident-related variables, fuzzy congestion features, GAN-enhanced features, and congestion labels.

To further evaluate the generalizability of the proposed framework beyond a single city, an additional external validation is conducted using a Lahore city, Pakistan, traffic dataset representing a different metropolitan traffic environment. The external dataset consists of 55,000 traffic observations collected from 30 ANPR monitoring cameras distributed across 20 geographic sectors during September 2025. The dataset includes the same traffic-flow, temporal, peak-hour, historical accident, accident propagation, fuzzy congestion, GAN-enhanced, and congestion-label features used in the Islamabad experiments to ensure a consistent evaluation protocol. The proposed APG-GRU framework and the baseline models have been tested on the Lahore dataset with the same four experimental scenarios and the experimental methodology remains unchanged. This further validation enables assessment of the framework’s robustness across different urban traffic environments and demonstrates that the proposed accident-aware congestion prediction framework can be adapted to cities with comparable ANPR-based traffic monitoring systems and historical accident information.

The features used in both datasets are presented in Table 1. The integrated datasets support four experimental scenarios designed to evaluate the individual and combined effects of fuzzy logic-based congestion labeling, historical accident integration, accident propagation modeling, and GAN-based class balancing. Traffic-flow and speed-related features characterize recurring congestion patterns, while accident-related and accident propagation features capture the influence of non-recurring traffic disruptions. Peak-hour indicators further represent temporal variations in traffic demand, enabling a comprehensive evaluation of the proposed APG-GRU framework under diverse traffic conditions.

### Data Preprocessing and Feature Engineering

The raw ANPR traffic data and historical accident records are converted into useful input variables through data preprocessing and feature engineering. The process began by loading both datasets and standardizing date and time features, and by extracting temporal information, including hour and weekend status, and peak-hour status using Algorithm 1. Outliers are detected, and zero speeds are corrected to ensure data quality. Date and time in the ANPR traffic data are used to integrate records into traffic-flow variables, and accident frequency, severity, fatalities, injuries, weather-related accidents, and vehicle involvement data in the accident data are used to create accident-related variables. These two data sets are then cross-referenced on date, hour, geographic sector, police station, and camera location. Congestion-related variables are generated using fuzzy logic, and the final congestion label is assigned using fuzzy logic; the integrated variables are added using GAN to balance the congestion representation. The most relevant variables for congestion prediction are selected using exploratory data analysis and correlation-based feature selection. The final integrated dataset comprises four groups of variables: ANPR traffic variables, peak-hour temporal variables, fuzzy logic variables, and historical accident-aware variables, which are also used in the four experimental scenarios of the results section. The set of dataset features is presented in Table 1 above.
**Algorithm 1:** Data Preprocessing and Feature EngineeringInput: Raw ANPR traffic dataset and accident dataset  Output: Cleaned and integrated dataset (D)Load the raw ANPR traffic dataset and accident dataset.Convert date and time fields into a standard datetime format.Extract temporal features, including hour, day of week, peak-hour status, and weekend status.Define peak hours as morning and evening high-demand periods: ({7, 8, 9, 16, 17, 18, 19}).Generate GAN-based integrated features to support balanced representation of congested traffic records.Clean the ANPR traffic records by correcting zero-speed values, removing invalid speed values and handling missing values using mean or mode imputation.Generate traffic-flow features from vehicle count, speed, violation information, speed drop, and speed variation.Clean the accidental records by removing duplicate, incomplete, and invalid records.Generate accident-aware features from accident frequency, accident severity, fatalities, injuries, weather-related incidents, and vehicle involvement information.Integrate ANPR traffic and accident records using date, hour, geographic sector, police station, and camera-location information.Apply fuzzy logic to generate fuzzy congestion scores and assign the final congestion label.Convert selected features into numeric format and apply exploratory analysis and correlation-based feature selection.Select the final feature groups, including ANPR traffic features, temporal and peak-hour features, accidental features, fuzzy congestion features, and GAN-based integrated features.Save the final cleaned and integrated dataset (D) for model training and evaluation.Return (D).

**Figure 1:**
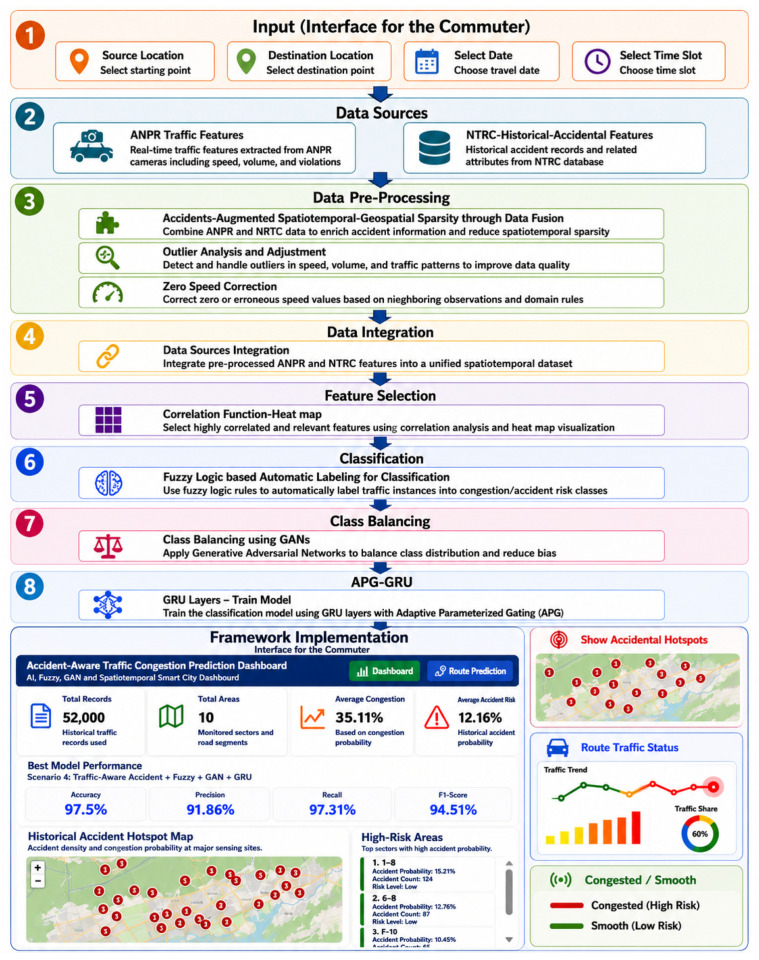
Process flowchart and architecture of the proposed APG-GRU framework.

Algorithm 1 summarizes the preprocessing and feature engineering procedure, while Figure 1 presents the complete APG-GRU workflow as a process flowchart.

## 4. APG-GRU Framework Methodology

The proposed APG-GRU framework is designed for accident-aware and peak-hour-based traffic congestion prediction using the cleaned and integrated traffic–accident dataset. To present the proposed procedure more clearly, the complete framework is illustrated through the process flowchart shown in Figure 1, while the detailed training and evaluation steps are provided in Algorithm 1. As shown in Figure 1, the APG-GRU framework starts with input information, including source location, destination location, date, and time slot. The data source layer integrates ANPR traffic features and historical accident features. The preprocessing stage performs accident-augmented spatiotemporal and geospatial data fusion, outlier analysis, adjustment, and zero-speed correction. The processed data are then integrated and passed through correlation-based feature selection. Fuzzy logic is applied to generate congestion labels, while GAN-based class balancing is used to improve congested-class representation. Finally, the GRU model is trained to predict traffic congestion status, and the trained APG-GRU framework is implemented through a dashboard for accident hotspot visualization and route-level smooth or congested traffic status.

The novelty of the proposed APG-GRU framework lies in the integration of historical accident-aware features, peak-hour traffic features, fuzzy congestion labeling, GAN-based class balancing, GRU-based temporal prediction, and dashboard-based visualization within a single congestion prediction pipeline. The proposed contribution is not the isolated use of existing tools, but their systematic integration to model both recurring and non-recurring congestion. Recurring congestion is represented through peak-hour and temporal traffic features, while non-recurring congestion is represented through historical accident-related features. Four experimental scenarios are designed to evaluate the individual and combined effects of traffic features, accident features, fuzzy labeling, and GAN-based class balancing. Scenario 4 represents the complete APG-GRU framework and demonstrates that incorporating historical accident information improves the quality of traffic congestion prediction. The complete training and evaluation procedure of the proposed APG-GRU framework is presented in Algorithm 2.
**Algorithm 2:** APG-GRU Framework Training and EvaluationInput: Cleaned and integrated traffic–accident dataset (D) Output: Scenario-wise predictions, evaluation metrics, confusion matrices, and best-performing APG-GRU scenarioLoad the cleaned and integrated dataset (D).Define the target label (y) as the congestion label, where (0) represents non-congested traffic and (1) represents congested traffic.Select the required feature groups from (D), including ANPR traffic features, peak-hour features, fuzzy congestion features, accident features, and GAN-based integrated features.Define four experimental scenarios using different combinations of traffic, accident, fuzzy, and GAN-based features.For each scenario, prepare the corresponding feature set (X_S).Convert selected features into numeric format and replace remaining missing values.Split the scenario dataset into training and testing sets.Normalize the training and testing feature sets.Apply GAN-based class balancing in the scenarios that include GAN-based integrated features.Reshape the data into GRU-compatible input format.Train the APG-GRU model for each scenario using the training data.Predict congested and non-congested traffic classes using the testing data.Evaluate each scenario using accuracy, congested precision, congested recall, congested F1-score, and confusion matrix values.Compare the four scenarios and select the best-performing scenario based on the highest congested F1-score, highest accuracy, and lowest false predictions.Return the scenario-wise results, confusion matrices, and final APG-GRU framework.

### 4.1. Accident Feature Construction

Road accident feature engineering is performed to show the effect of historical road accidents on traffic congestion. The accident data is used to obtain accident-related data, which is then merged with the ANPR traffic data. This step intends to collect the frequency, severity, and spatial occurrence of road accidents that could lead to a loss of roadway capacity and an interruption of vehicle movements, and cause non-recurring congestion. Several features related to the accidents are extracted for each traffic observation: local accident count, incident count, area-level accident count, average number of vehicles involved, accident severity score, fatality count, injury count, weather-related incident count, and Accident Risk Score. These features are the number of accidents, the severity of the accidents, and the exposure to the traffic location at which the accidents occurred. The local accident count is the number of accident events in the same geographic sector as the location of the ANPR camera, such as in the same street, lane, and road, as indicated in Equation (1).

(1) $$Accident\text{\_}Count\text{\_}Local\;(s)=N_{accidents}\;(s)$$

where s is the geographic sector associated with the ANPR camera location, and $N_{accidents}\;(s)$ demonstrates the total number of accidents recorded in that sector.

The area-level accident count presents the number of accident events recorded in an area-level traffic zone consisting of nearby sectors or camera locations, as presented in Equation (2).

(2) $$Area\text{\_}Accident\text{\_}Count\;(a)=N_{accidents}\;(a)$$

In Equation (2), a represents an area-level traffic zone consisting of nearby sectors or camera locations, and $N_{accidents}\;(a)$ represents the total number of accidents recorded in that area.

To provide a clear accident impact indicator, the Accident Risk Score is calculated by combining normalized accident-related features. The accident-related features have different measurement scales; min–max normalization is first applied, as shown in Equation (3).

(3) $$X_{i\;j}^{norm}=\frac{X_{j\;i}-{\;X}_{j}^{min}}{X_{j}^{max}-X_{j}^{min}}\;$$

In Equation (3), $X_{j\;i}$ represents the value of accident-related feature j for traffic record i, ${\;X}_{j}^{min}$ and $X_{j}^{max}$ represent the minimum and maximum values of feature *j*, and $X_{i\;j}^{norm}$ represents the normalized value of the accident-related feature. After normality, the Accident Risk Score is calculated as the average of the normalized accident-related features, as shown in Equation (4).

(4) $${Accident\text{\_}Risk\text{\_}Score}_{i}=\frac{1}{k}\;\sum_{j=1}^{k}X_{i\;j}^{norm}$$

In Equation (4), *k* represents the total number of accident-related features used in the calculation. These features consist of local accident count, incident count, area-level accident count, accident severity score, fatality count, injury count, weather-related incident count, and average number of vehicles involved. A higher ${Accident\text{\_}Risk\text{\_}Score}_{i}$ indicates a higher historical accident impact for the corresponding traffic record. The extracted accident-related features are then integrated with traffic-flow features, fuzzy congestion scores, peak-hour indicators, and GAN-based integrated features. This integration enables the APG-GRU framework to learn recurring congestion patterns associated with regular traffic demand and non-recurring congestion patterns associated with historical road accidents.

### 4.2. Accident Propagation Modeling

Considering the propagation effect of road accidents on traffic congestion, accident information is additionally modeled by spatiotemporal propagation features. Simple count and severity variables indicate whether an accident has been recorded in a location but are not complete indicators of how the impact of the accident may ripple outward to adjacent road segments and/or extend over time. The proposed APG-GRU framework therefore also incorporates an accident propagation representation which is based on the spatial, temporal, and accident severity.

For each ANPR traffic record *i*, accident events *e* occurring near the corresponding camera location and within the relevant time period are considered. The temporal difference between a traffic record and an accident event is defined as:

(5) $$∆t_{ie}\;=t_{i}\;-t_{e}\;$$

In Equation (5), $t_{i}$ is the timestamp of traffic record *i*, and $t_{e}$ is the timestamp of accident event *e*. Only accident events occurring before or close to the traffic observation are considered for propagation modeling.

The spatial distance between the ANPR camera location and the accident location is represented as:

(6) $$d_{ie}\;=\;Distance\;(l_{i}\;-l_{e})$$

In Equation (6) $l_{i}$ represents the location of the ANPR camera for traffic record *i*, and $l_{e}$ represents the location of accident event *e*.

The spatiotemporal accident propagation score is calculated as:

(7) $${Accident\text{\_}Propagation\text{\_}Score}_{i}=\;\sum_{e=1}^{m}\;{Severity}_{e}\;\times \exp\;\left(-\frac{d_{ie}}{\lambda }\right)\;\times \exp\left(-\frac{{∆t}_{ie}}{\tau }\right)$$

In Equation (7), *m* is the number of accident events considered for traffic record *i*, ${Severity}_{e}$ represents the severity weight of accident event *e*, $d_{ie}$ represents the spatial distance between the accident and the camera location, ${∆t}_{ie}$ represents the temporal difference between the accident and traffic record, *λ* is the spatial decay parameter, and τ is the temporal decay parameter. The exponential decay terms reduce the influence of accidents that are farther away in space or time.

This propagation score enables the model to capture the indirect effect of accidents on nearby road segments and later traffic observations. Therefore, accident modeling in the revised APG-GRU framework is not limited to accident count, severity, fatalities, and injuries; it also includes a propagation-aware representation of accident influence. The final accident feature group includes local accident count, area-level accident count, accident severity score, fatality count, injury count, weather-related incident count, average vehicles involved, Accident Risk Score, accident proximity, temporal accident influence, and accident propagation score. This improves the ability of the framework to model non-recurring congestion caused by accident-induced traffic disturbance.

### 4.3. Peak-Hour Representation

The peak travel periods, when many vehicles are traveling on the road network, are also important factors for recurring traffic congestion. In smart cities, heavy traffic in the city is common in the morning and evening hours, and around offices, educational institutes, commercial areas, and major intersections. To achieve this, the APG-GRU framework incorporates temporal features such as Hour, Day_of_Week, Is_Peak_Hour, and Is_Weekend. The feature Is_Peak_Hour is coded as a binary variable; 1 = peak hour; 0 = non-peak hour. Peak-hour information is needed because traffic congestion is not only dependent on the volume of traffic but also on when the traffic is demanded. A medium traffic incident during peak hours can produce a higher congestion impact on traffic congestion whereas the same incident during low traffic demand has a low impact on traffic. Thus, peak-hour indicators can be used to give context to the congestion event and help the model separate recurring congestion due to a regular traffic demand from non-recurring congestion due to road accident events, thereby enhancing the performance of the model in predicting congestion [25].

### 4.4. Fuzzy Logic Based Labeling

The fuzzy labeling technique is used to label traffic speed observations using a fuzzy inference approach rather than a fixed speed threshold. To label congestion in the proposed APG-GRU framework, the average speed of each ANPR traffic observation is adopted as the primary input. Low speed values are considered as congested traffic, high speed values as smooth traffic, and intermediate speed values as transitional traffic. The average speed of each traffic record is mapped into three fuzzy speed states representing low, medium, and high traffic conditions. This process produces a fuzzy congestion score for each traffic observation. Records with a stronger association to the low-speed state are assigned a higher fuzzy congestion score, whereas records associated with higher speeds receive a lower congestion score. The fuzzy congestion score is subsequently used to assign the final binary congestion label. If the fuzzy congestion score reaches the predefined congestion-labeling threshold, the record is labeled as Congested; otherwise, it is labeled as Smooth.

The two significant outputs of this technique are Fuzzy_Congestion_Score and Congested_Label. The Fuzzy_Congestion_Score represents the degree of congestion intensity, while Congested_Label represents the final binary class used for model training and testing. This labeling strategy reduces the boundary problem associated with hard thresholding because traffic conditions near the transition between smooth and congested states are represented more flexibly. The same fuzzy labeling procedure is consistently applied to all traffic observations before model development, ensuring identical labeling criteria across all four experimental scenarios and enabling fair performance comparison. Similar findings have been reported in recent traffic management studies [18,19], where fuzzy logic has been demonstrated to be an effective model of uncertain and saturated traffic states.

The congestion-labeling procedure is completed before the model training stage. The Fuzzy_Congestion_Score is generated from traffic speed observations and represents the continuous degree of congestion intensity, whereas Congested_Label represents the final binary target class used for congestion prediction. The predictor variables consist of traffic-flow characteristics, temporal indicators, accident-related features, Fuzzy_Congestion_Score, and the GAN-balanced training samples where applicable. Following the congestion-labeling process, the complete dataset is partitioned into training and testing subsets. GAN-based balancing is subsequently applied only to the training subset in Scenarios 2 and 4, while the testing subset remains unchanged. The balanced training data are then used to train the proposed GRU model, whereas Scenarios 1 and 3 are trained using the original training data without GAN balancing. The sequential implementation procedure is followed in all four experimental scenarios, ensuring a fair, transparent and reproducible assessment of the proposed APG-GRU framework.

### 4.5. GAN-Based Balancing

The integrated traffic congestion dataset is imbalanced, with non-congested traffic observations being more frequent than congested observations. The final dataset contained 40,506 non-congested records and 11,494 congested records. This imbalance could lead the learning model to favor the majority non-congested class and reduce its ability to correctly detect congested traffic cases. Therefore, GAN-based class balancing is applied to improve the representation of the minority congested class during model training.

The balancing process is performed after data preprocessing, fuzzy labeling, feature selection, and train–test splitting. To avoid data leakage, GAN-based balancing is applied only to the training data, while the testing data are kept unchanged in their original class distribution. The training data are first divided into majority non-congested samples and minority congested samples. The GAN model is trained on the minority congested-class feature space to learn the distribution of congested traffic records. During adversarial learning, the generator produces synthetic congested traffic samples, while the discriminator simultaneously learns to distinguish between real and generated samples. Through this iterative optimization process, the generator progressively improves its ability to produce realistic minority-class samples that preserve the statistical characteristics of the original congested traffic records. After training, the generator is used to generate additional synthetic congested samples. These generated samples are then combined with the real training samples to create a more balanced training set. The resulting balanced training dataset is subsequently used to train the GRU-based congestion prediction model, whereas the testing dataset remains unchanged to provide an unbiased evaluation of model performance.

GAN balancing is used only in Scenarios 2 and 4. Scenario 2 is designed to evaluate the contribution of GAN-based class balancing in the absence of historical accident-aware features, whereas Scenario 4 evaluates the combined contribution of GAN-based balancing and historical accident-aware features. In contrast, Scenarios 1 and 3 use the original imbalanced training dataset without GAN balancing. This experimental design enables the individual and combined effects of GAN-based balancing and historical accident-aware feature engineering to be systematically evaluated. The purpose of GAN balancing is not to replace real traffic observations but to improve minority-class representation during training and reduce the prediction bias toward the non-congested class. The final model evaluation is performed on the unchanged test set using accuracy, congested precision, congested recall, congested F1-score, and confusion matrix analysis. The balanced training dataset obtained after GAN-based augmentation is directly used as the input for GRU model training in Scenarios 2 and 4, while the original testing dataset is retained for final performance evaluation.

### 4.6. Model Selection and Experimental Scenarios

The APG-GRU framework uses a Gated Recurrent Unit (GRU) network as the key prediction model. Due to the temporal dependencies of traffic, it is decided that GRU is a suitable choice for development. This means that the current traffic situation depends on the previous traffic situation, accident occurrences, and peak-hour demand patterns. GRU has fewer parameters and lower computation complexity than traditional recurrent neural networks, but it can still provide a good learning ability for sequence traffic data. Four experimental scenarios are created to assess the role of the features of accidents and class balancing using GAN. The same GRU prediction architecture is used across each scenario with the components of the framework gradually added. This design allows for the systematic evaluation of the effect of fuzzy labeling, accident integration and GAN balancing on the performance of the congestion prediction.

Experimental scenarios used in the APG-GRU Framework are presented in Table 2. Scenario 1 serves as the baseline model using traffic and temporal features with fuzzy congestion labeling. Scenario 2 evaluates the contribution of GAN-based class balancing. Scenario 3 investigates the impact of accident-related information on congestion prediction. Finally, Scenario 4 represents the complete proposed APG-GRU framework, combining traffic features, peak-hour indicators, accident information, fuzzy congestion labeling, GAN balancing, and GRU sequence learning. The performance of all scenarios is compared using accuracy, congested precision, congested recall, congested F1-score, and confusion matrix analysis. The Random Forest, XGBoost, LSTM, and GRU models were selected to evaluate machine learning and DL approaches [26] for traffic congestion prediction, with GRU adopted as the proposed prediction model.

The proposed APG-GRU consists of two feature groups: one for recurring congestion and another for non-recurring congestion, though these groups are integrated. Recurring congestion is congestion that happens repeatedly at regular times of traffic demand, particularly in the morning and evening peak hours. It is encoded with temporal and peak-hour features such as hour, day of week, weekend, or peak-hour status. This capability enables the framework to recognize the normal congestion patterns for periods of the day when travel demand is heavy. Non-recurring congestion is any congestion that results from an unusual event such as road crashes. It is modeled by different accident-related features from the accident database that are typically associated with accidents, such as local accident count, incident count, area-level accident count, accident severity score, fatality count, injury count, weather-related incident count, average number of vehicles involved, or Accident Risk Score. These accident-aware features are combined with ANPR traffic observations, thereby enabling the model to learn how traffic conditions in the past affect congestion formation. The classification task in this study is an online classification of traffic condition for a given route, date, and time slot, using the trained APG-GRU model to classify the chosen traffic condition as either smooth or congested.

### 4.7. GRU-Based Congestion Prediction Model

The proposed APG-GRU framework’s final stage, prediction, is based on the Gated Recurrent Unit (GRU) network. The GRU proposed by [27] is a variant of the recurrent neural networks (RNNs) that can capture long-term temporal dependency with low computational complexity compared with the conventional recurrent architectures. GRU is appropriate for learning sequential traffic behavior because it is a problem that changes over time and is influenced by the previously observed traffic conditions, accident occurrences, and the traffic demand pattern during the peak hour. There are two gates in a GRU cell: the update gate and reset gate. The update gate regulates the amount of information carried over from the previous time step, and the reset gate regulates the amount of information that is erased from the previous time step. These gates allow the network to learn the short-term traffic patterns and the long-term traffic patterns effectively. The update gate is computed as Equation (8):

The update gate controls how much of the past information to pass to the future:

(8) $$z_{t}=\;\sigma \;(W_{z}\;x_{t}+\;U_{z}\;{\;h}_{t-1}+b_{z})$$

The reset gate determines how much of the past information to forget in Equation (9):

(9) $${\;r}_{t}=\;\sigma \;(W_{r}\;x_{t}+\;U_{r}\;{\;h}_{t-1}+b_{r})$$

The candidate hidden state combines the current input with the reset hidden state:

(10) $${\tilde{h}}_{t}=\;tanh\;(W_{h}\;x_{t}+U_{h}\;{(r}_{t}⨀h_{t-1})+b_{h})$$

The final hidden state updates the previous state with the candidate:

(11) $$h_{t}=(1-z_{t})⨀h_{t-1}\;+z_{t}⨀{\tilde{h}}_{t}$$

**Figure 2:**
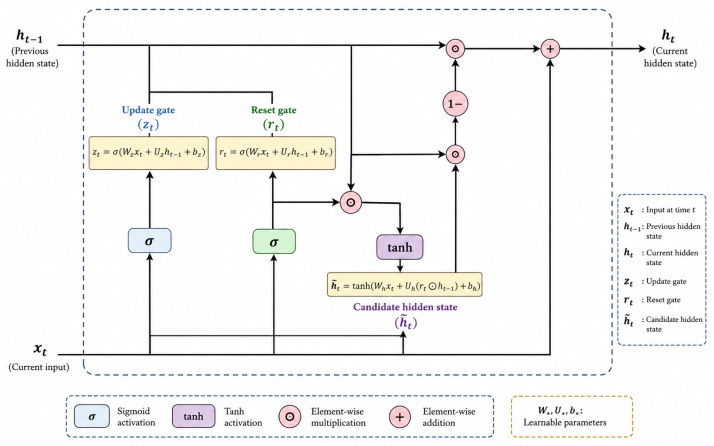
Internal architecture of the GRU cell used in the proposed APG-GRU framework [27].

Update gate: Where the previous hidden state and the candidate hidden state are updated together. In Equations (10) and (11), *x_t_* is the feature vector at time (*t*) for the input, *h_t_*_−1_ is the hidden state at the previous time step, (*W*) and (*U*) are matrices of trainable weights, (*b*) are bias terms, σ is the sigmoid activation function, and ⊙ is the element-wise multiplication. The traffic-flow features, peak-hour indicators, accident-related variables, fuzzy congestion scores, and GAN-balanced samples are provided to the GRU model in the proposed framework based on the experimental scenarios. The model is trained on temporal relationships between these features and classifies the traffic observation as a Congested class or a Non-Congested class. GRU can capture the sequential nature of traffic patterns, which is suitable for modeling recurring congestion (due to peak-hour traffic) and non-recurring congestion (due to road accidents). The internal architecture of the GRU cell used in the APG-GRU framework is shown in Figure 2.

In Figure 2, W∗, U∗, and b∗ denote the learnable weight matrices and bias vectors associated with the GRU gates, where the asterisk (*) represents the corresponding gate (update, reset, or candidate hidden state). Before GRU training, the input data are preprocessed using the proposed APG-GRU framework, including feature engineering, fuzzy logic-based congestion labeling, train–test partitioning, and GAN-based balancing where applicable. The resulting training dataset is used to learn the temporal relationships among traffic-flow characteristics, temporal indicators, accident-related features, and fuzzy congestion scores. For Scenarios 2 and 4, the GRU model is trained using the GAN-balanced training dataset, whereas Scenarios 1 and 3 utilize the original training dataset without class balancing. After model training, the learned GRU model is applied to the unchanged testing dataset to predict the congestion class of each traffic observation. The predicted congestion labels are then compared with the ground-truth labels to evaluate the model using accuracy, congested precision, congested recall, congested F1-score, and confusion matrix analysis. This implementation procedure is consistently followed across all four experimental scenarios to ensure a fair and reproducible evaluation of the proposed APG-GRU framework.

### 4.8. Evaluation Metrics

Accuracy, congested precision, congested recall, and congested F1-score are used to assess the performance of the proposed APG-GRU framework presented in Table 3. These metrics are chosen since the main goal of the framework is to correctly classify traffic congestion, with a minimum number of misclassifications. Accuracy is the overall percentage of correctly classified traffic observations. Congested precision is the ability to accurately predict the congestion of the observations. Congested recall is a metric used to evaluate the model’s effectiveness in detecting real congestion events, which is crucial in ITS where the absence of congestion can have a significant impact on traffic management and route planning. The F1-score is used as a single performance measure and is a balance of precision and recall, particularly when the congested class is a smaller percentage of the data. The following metrics are computed for all four experimental scenarios to evaluate the effect of accident integration, peak-hour features, fuzzy labeling, and class balancing using GAN.

The evaluation metrics used for scenario comparison are presented in Table 3. In this study, True Congestion (TC) represents congested traffic correctly predicted as congested, while True Smooth (TS) represents smooth traffic correctly predicted as smooth. False Congestion (FC) refers to smooth traffic incorrectly predicted as congested, whereas False Smooth (FS) refers to congested traffic incorrectly predicted as smooth. These metrics provide a comprehensive assessment of the proposed APG-GRU framework and enable a fair comparison among the four experimental scenarios.

### 4.9. Statistical Reliability and Modern Baseline Evaluation

To improve the reliability of the experimental evaluation, repeated-run analysis is performed by using five independent random seeds: 11, 22, 33, 44, and 55. For each model, accuracy, congested precision, congested recall, and congested F1-score are reported as the mean value, standard deviation, and 95% confidence interval. In addition, paired statistical testing is performed to compare the proposed APG-GRU framework with the baseline models. A paired t-test is used because the compared models are evaluated under the same repeated-run setting. A significance level of *p* < 0.05 is considered statistically significant. To further strengthen the comparative evaluation, two modern spatiotemporal traffic prediction baselines are included: Spatio-Temporal Graph Convolutional Network (STGCN) and Diffusion Convolutional Recurrent Neural Network (DCRNN). For these graph-based models, camera locations are treated as graph nodes.

## 5. Results and Discussion

### 5.1. Correlation Analysis of Traffic and Historical Accident Features

**Figure 3:**
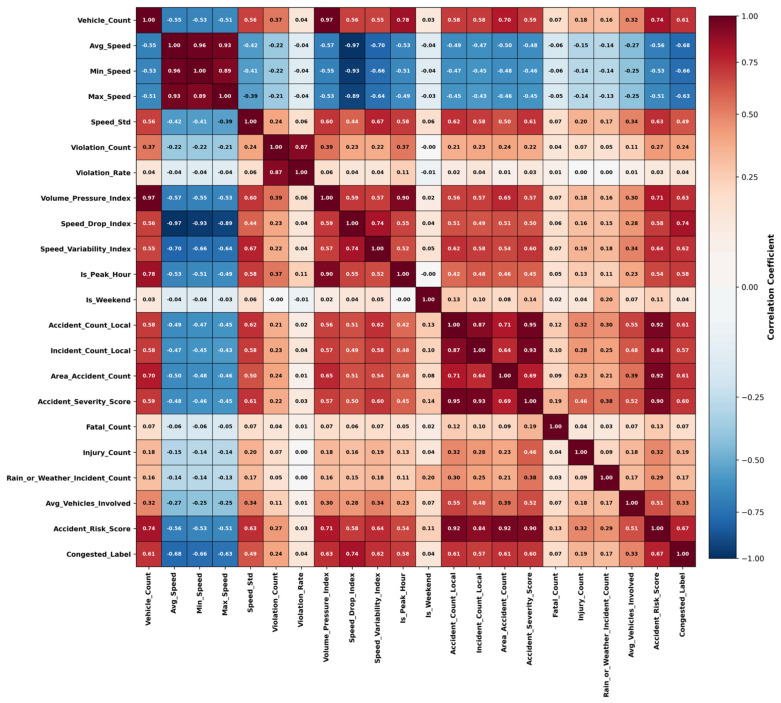
Correlation analysis of traffic and historical accident features.

A correlation heatmap of the traffic variables, the features in the accident record, and the congestion label is shown in Figure 3. From the analysis, several important relationships can be observed. Vehicle Count shows a positive correlation with congestion with the number of vehicles rising with congestion. Average Speed, Minimum Speed and Maximum Speed are highly negatively correlated with congestion, which validates the notion that traffic speeds reduce significantly during congestion. Likewise, the Speed Drop Index and Speed Variability Index show positive correlations with congestion as rapid deceleration is often linked with congestion events. Of the accident-related features, Accident Count Local, Area Accident Count, accident severity score, and Accident Risk Score show positive correlations with congestion. The results show that if the frequency of accidents and the severity of accidents increase, so will the severity of traffic congestion. There are also positive relationships between peak-hour indicators and congestion, indicating the impact that morning and evening peak-hour traffic has on road network performance. The overall correlation analysis identifies the traffic volume and demand for travel during peak hours as recurring factors, while identifying accidents and incident severity as non-recurring factors, which affect traffic congestion.

### 5.2. Scenario-Wise Performance

The scenario-wise performance comparison of the proposed APG-GRU framework is presented in Table 4. Scenario 1, which uses ANPR traffic features, peak-hour features, fuzzy labeling, and GRU prediction, provides the baseline performance with an 86.50% accuracy, a 64.49% congested precision, an 86.64% congested recall, and a 73.94% congested F1-score. In Scenario 2, the addition of GAN-based class balancing improves the accuracy to 90.50%, congested precision to 73.02%, congested recall to 90.45%, and congested F1-score to 80.80%. This improvement shows that GAN-based balancing helps reduce the effect of class imbalance and improves congested-class learning. Scenario 3 further improves the results by adding accident-aware features, achieving a 94.50% accuracy, an 82.91% congested precision, a 94.62% congested recall, and an 88.38% congested F1-score. This confirms that historical accident features help the model capture non-recurring congestion caused by road incidents. The best performance is achieved in Scenario 4, where ANPR traffic features, accident features, peak-hour features, fuzzy labeling, GAN-based balancing, and GRU prediction are combined. Scenario 4 achieved a 97.50% accuracy, a 91.86% congested precision, a 97.31% congested recall, and a 94.51% congested F1-score. These results confirm that the complete APG-GRU framework provides the most reliable congestion prediction performance among all four scenarios.

The comparative performance of Random Forest, XGBoost, and LSTM across the four experimental scenarios is presented in Table 5. The results show that the inclusion of accident-aware features generally improves the performance of the baseline models, especially in Scenario 3 and Scenario 4. For Random Forest, the congested F1-score increased from 85.2% in Scenario 1 to 91.5% in Scenario 3 and Scenario 4. XGBoost also improved from 85.8% in Scenario 1 to 91.3% in Scenario 3 and 91.2% in Scenario 4. Similarly, LSTM improved from 85.3% in Scenario 1 to 91.0% in Scenario 3 and 90.5% in Scenario 4. These results indicate that accident-aware features are useful.

Although the baseline models achieved robust results, the proposed APG-GRU framework showed the best overall performance in Scenario 4. Compared with Random Forest, XGBoost, and LSTM, APG-GRU achieved higher accuracy and a higher congested F1-score. Random Forest produced high congested precision values, while XGBoost and LSTM achieved high congested recall values; however, APG-GRU provided the best balance between congested precision and congested recall. This balance is important for traffic congestion prediction because the model must correctly detect actual congestion while also reducing false congestion alerts. Therefore, the results demonstrate that integrating historical accident features, peak-hour features, fuzzy congestion labeling, GAN-based class balancing, and GRU-based temporal learning improves accident-aware traffic congestion prediction.

The repeated-run statistical comparison of the proposed APG-GRU framework with machine learning, deep learning, and graph-based traffic prediction baselines is presented in Table 6. The results show that APG-GRU achieved the highest overall performance, with a 97.375% mean accuracy and a 94.911% mean congested F1-score. The confidence intervals also demonstrate that APG-GRU produced stable and consistent results across repeated runs. Among the baseline models, XGBoost achieved the strongest congested F1-score, followed by Random Forest. The graph-based models STGCN and DCRNN achieved better congested recall than the basic recurrent models, showing their ability to capture spatiotemporal traffic dependencies. However, APG-GRU achieved the best overall balance between congested precision and congested recall. The confidence intervals indicate that APG-GRU consistently achieved superior performance across repeated runs, demonstrating the stability, reliability, and robustness of the proposed framework.

### 5.3. Confusion Matrix Results

**Figure 4:**
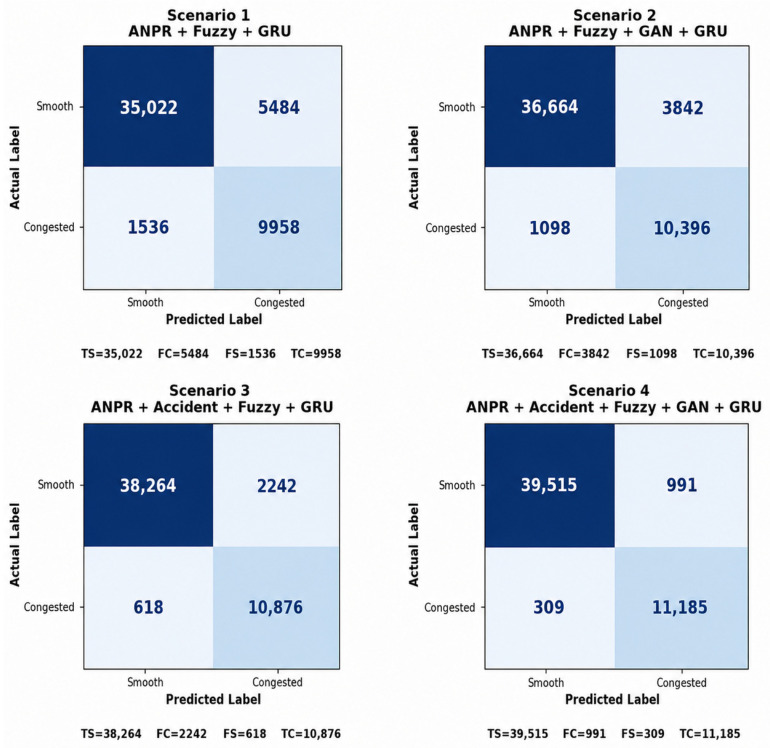
Confusion matrices of the four APG-GRU experimental scenarios.

The confusion matrices of the four APG-GRU experimental scenarios are shown in Figure 4. The number of correctly classified traffic records for each class (Smooth and Congested) is shown, and not the percentage share of evaluation metrics. True Smooth (TS) denotes the number of smooth traffic flows correctly identified as smooth, False Congestion (FC) denotes the number of smooth traffic flows incorrectly identified as congested, False Smooth (FS) denotes the number of congested traffic flows incorrectly identified as smooth, and True Congestion (TC) denotes the number of congested traffic flows correctly identified as congested. The greatest number of false predictions is made with scenario 1 (5484 FC and 1536 FS). Using the GAN-based balancing in Scenario 2 lowered the number of false predictions to 3842 FC and 1098 FS. Including the accident-aware features further enhanced the classification results as shown in the third scenario (FC: 2242, FS: 618). The highest confusion matrix performance is obtained by Scenario 4, having a total of 39,515 TS, 991 FC, 309 FS, and 11,185 TC. It is shown that by combining ANPR traffic features, accident-aware traffic features, fuzzy labeling, GAN-based balancing, and GRU prediction, the complete APG-GRU framework is able to reduce false congestion alerts and improve the detection of real congestion traffic cases.

### 5.4. External Validation and Generalizability Analysis

To evaluate the generalizability of the proposed APG-GRU framework beyond the primary study area, an additional external validation is conducted using the Lahore traffic dataset. The external dataset consists of traffic flow, historical accident, peak hour, fuzzy congestion, and GAN-enhanced features that are compatible with the proposed framework. The same four experimental scenarios and evaluation methodology used for the Islamabad dataset are applied to the Lahore dataset to assess the robustness and transferability of the proposed model under a different urban traffic environment. The results are summarized in Table 7 and Table 8.

The external validation results of the proposed APG-GRU framework on the Lahore city dataset under four experimental scenarios are presented in Table 7. Similar to the results obtained on the Islamabad dataset, the prediction performance consistently improved as additional accident-aware features and GAN-based balancing were incorporated into the framework. Scenario 1 achieved an accuracy of 91.78% and a congested F1-score of 83.83%, while the inclusion of GAN-based balancing in Scenario 2 slightly improved the overall performance. Incorporating historical accident-aware features in Scenario 3 produced a substantial improvement, increasing the accuracy to 95.29% and the congested F1-score to 90.14%. The highest performance is achieved in Scenario 4, where the combined use of accident-aware features and GAN-based balancing resulted in a 95.41% accuracy and a 90.32% congested F1-score. The result findings determine the ability of the proposed APG-GRU to consistently predict traffic state in another urban traffic environment, indicating the effectiveness and applicability of the framework in further areas beyond the primary study area.

The performance of Random Forest, XGBoost, and LSTM across the four experimental scenarios using the Lahore city dataset is compared in Table 8. All baseline models showed improved performance in Scenarios 3 and 4 after incorporating historical accident-aware features and GAN-based balancing. Among the baseline models, Random Forest achieved the best overall performance with accuracy and congested F1-score, while XGBoost and LSTM also demonstrated notable improvements. However, the proposed APG-GRU framework in Table 7 consistently achieved superior overall congestion prediction performance across the corresponding scenarios, demonstrating that the integration of historical accident-aware features, fuzzy logic-based labeling, and GAN-based balancing provides a more effective representation of complex traffic conditions. These results further confirm the robustness and generalizability of the proposed framework across different urban traffic environments.

### 5.5. Visualization Dashboard of APG-GRU Framework

**Figure 5:**
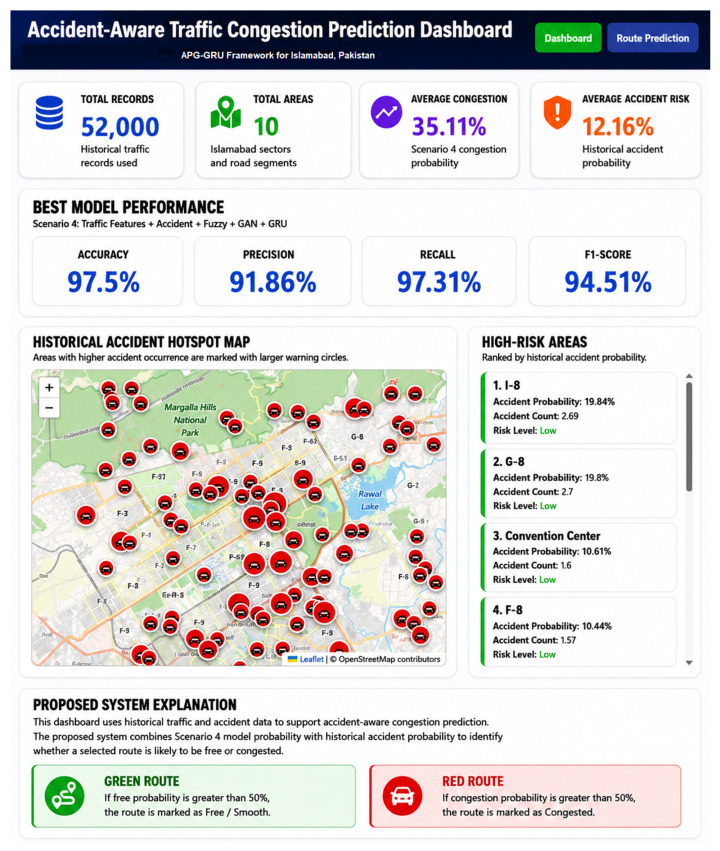
APG-GRU dashboard for accident hotspot visualization.

The evolved APG-GRU dashboard is a practical implementation of the proposed traffic congestion prediction framework by incorporating accident-related information. It presents an overview of integrated data that consists of 52,000 traffic records, 10 monitored sector areas, a 35.11% traffic congestion probability on average, and a 12.16% traffic accident risk on average. The dashboard also shows the best model performance in Scenario 4, which is a 97.5% accuracy, 91.86% precision, 97.31% recall, and 94.51% F1-score. The accident hotspot map reflects the occurrence of historical accidents, and the high-risk area panel indicates the sectors ranking based on the historical accident probability and number of accidents presented in Figure 5. I-8, G-8, F-8 and Conventional Center are official sector names in Islamabad, Pakistan, used as geographic identifiers. This demonstrates the ability of the dashboard to merge these disparate functions of congestion prediction, accident hotspot visualization, and route-risk interpretation into a single decision-support interface to benefit traffic managers and commuters.

**Figure 6:**
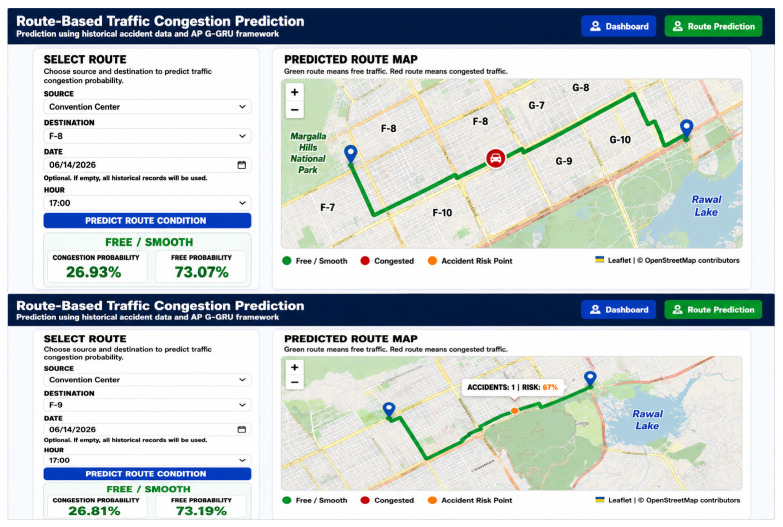
Route-based APG-GRU dashboard for congestion prediction and accident-aware route assessment.

The route-based traffic congestion prediction dashboard is tested with two cases of route prediction presented in Figure 6. The sector labels (e.g., F-8, F-9, G-7, and G-8) are official administrative sector names of Islamabad, Pakistan, used as geographic identifiers. The same user decided the source location, destination, date, and time for both tests, and the dashboard predicted the route status based on the historical record of accidents and the APG-GRU framework. The first test predicted the route from the Convention Center to F-8 as a Smooth route with a congestion probability of 26.93% and a smooth-flow probability of 73.07%. The second test predicted the route between the Convention Center and F-9 as Smooth with a probability of congestion of 26.81% and a probability of smooth flow of 73.19%. Accident risk points are also shown along the routes selected in the predicted route maps, which indicate that the dashboard can be used not only to predict congestion but also to assess the route regarding accidents. The results show the applicability of the APG-GRU framework for traffic monitoring and decision support for routes.

### 5.6. Discussion

The results of this study show that the proposed APG-GRU framework is successful in traffic congestion prediction for smart city infrastructure. The result of progressive improvement from Scenario 1 to Scenario 4 demonstrates that the combination of accident information, fuzzy logic labeling and class balancing using GAN improves congestion prediction performance. The overall best performance is obtained by Scenario 4, in which all relevant traffic and accident data are shared in a common prediction framework. The traffic variables represent typical traffic conditions, and the accident variables represent unexpected traffic disruptions that may cause non-recurring congestion. The integration of these two data sources enables the APG-GRU framework to gain a more comprehensive understanding of the factors that impact traffic flow. Class imbalance is also a factor reduced by GAN balancing. Congested traffic is less common than smooth traffic in real-world traffic datasets. The model can learn the congestion patterns better by using the samples generated using GAN, which leads to better classification results.

To address the uncertainty in traffic states, fuzzy logic labeling further improved the quality of prediction. Fuzzy labeling is used in place of fixed thresholds to give a more realistic representation of congestion levels, consequently enhancing the learning ability of the GRU model. The results are consistent with earlier studies on traffic prediction, which presented positive results based on deep learning and historical accident-aware prediction techniques. But the proposed framework builds on existing works by embedding the information of accidents, peak-hour indicators, fuzzy labeling, GAN balancing, and GRU learning in a single architecture. This integration provides an improved congestion prediction compared to the traditional traffic-only methods [16,25,28,29].

The web-based dashboard also shows the applicability of the proposed framework for APG-GRU in the context of Smart City Islamabad. It displays accident hotspot visualization, congestion-risk information, and performance indicators for Scenario 4 in one interface as shown in Figure 5. The detailed results have already been presented, so the dashboard is more of a high-level description of how the proposed framework can be translated to a decision-support tool for monitoring high-risk areas, assessing road conditions, and supporting intelligent traffic management.

The results show that the proposed APG-GRU framework improves congestion prediction by integrating traffic-flow features, peak-hour information, accident-aware features, GAN-based class balancing, and GRU-based learning. The improvement from Scenario 1 to Scenario 3 confirms that accident-related features provide useful information for identifying non-recurring congestion, while the improvement in GAN-based scenarios shows that class balancing helps the model learn congested traffic cases more effectively. The confusion matrix results further show that the complete APG-GRU framework reduces both false congestion alerts and missed congestion cases, which is important for practical traffic management.

APG-GRU significantly outperformed machine learning, recurrent deep learning and graph-based baselines, as it incorporates accident-aware feature engineering with temporal learning. However, the study has some limitations. The experiments are based on one city and one selected observation period, so the exact results may vary in other cities, seasons, or road networks. The quality of accident-aware features also depends on the completeness and accuracy of accident records. In addition, the graph-based baselines are limited by the lack of explicit road network adjacency information.

## 6. Conclusions

This study presents the APG-GRU framework for traffic congestion prediction using Safe City Islamabad ANPR data and incorporating historical accident-related information. The proposed framework integrates ANPR traffic features, peak-hour indicators, fuzzy logic-based congestion labeling, historical accident-related features, GAN-based class balancing, and GRU sequence learning. The APG-GRU framework aims to improve congestion prediction by considering both non-recurring congestion caused by road accidents and recurring congestion associated with peak-hour traffic demand. Four experimental scenarios were designed to evaluate the individual and combined effects of historical accident integration and GAN-based balancing. The experimental results presented a progressive improvement in prediction performance from Scenario 1 to Scenario 4. Scenario 1 (ANPR + Fuzzy + GRU) achieved an accuracy of 86.50% and a congested F1-score of 73.94%, whereas Scenario 2 (ANPR + Fuzzy + GAN + GRU) achieved a higher congested F1-score of 80.80% through GAN-based class balancing. The addition of the accident-related information (Scenario 3: ANPR + Accident + Fuzzy + GRU) further improved the performance with an F1-score of 88.38%. The best results are obtained for Scenario 4 for the APG-GRU framework, with an accuracy of 97.50%, a congested precision of 91.86%, a congested recall of 97.31%, and a congested F1-score of 94.51%. The outcomes indicate that the features used in the accidents have good predictive power for traffic congestion forecasting. Moreover, the peak-hour indicators are able to capture the recurring congestion well, fuzzy logic labeling enhances the representation of the congestion, and GAN balancing tackles the class imbalance problem. These ingredients make it possible for the proposed framework to correctly detect congestion conditions and widely outperform simpler traffic-only prediction models. In terms of implementation, the proposed framework could prove to be useful for ITS in providing more accurate congestion forecasts, helping in route planning, minimizing travel delays, and better managing traffic decisions. The framework provides a useful and effective decision-support tool for Safe City Islamabad to keep an eye on traffic situations and to act proactively when traffic demand or road accidents induce congestion. The external validation results on the Lahore traffic dataset further confirm the robustness and generalizability of the proposed APG-GRU framework beyond the primary study area, supporting its applicability to other urban traffic environments. Furthermore, a web-based interface is also designed for the APG-GRU framework, which visualizes the accident hotspots and the traffic status at the route level (green routes standing for smooth traffic-flow and red routes standing for congested traffic flow conditions), showing the potential of practical deployment of the proposed system in the context of smart city traffic management.

Future work will investigate multi-city validation, longer observation periods, integration of real-time IoT and connected vehicle data, adaptive accident propagation modeling, and graph-based deep learning architectures to further improve scalability and deployment in heterogeneous urban environments.

## Figures and Tables

**Table 1:** ANPR traffic and historical accident dataset features.

S.No.	ANPR Traffic_Feature	Data_Type	S.No.	Accidental_Feature	Data_Type
1	ID	int64	1	Reporting Time	object
2	Date_Time	object	2	Crash Time	object
3	Date	object	3	Case Nature	object
4	Camera_ID	int64	4	Level1	object
5	Camera_Ip	Int64	5	Total Persons	float64
6	District	object	6	Location	object
7	Reg_No	object	7	Case Description	object
8	Voilation	object	8	Latitude	float64
9	Speed	int64	9	Longitude	float64
10	Geographic_Sector	object	10	Location	object
11	Camera_Name	object	11	Severity of Crash	object
			12	Weather	object
			13	Road Type	object
			14	No. of Vehicles Involved	float64
			15	Type of Collision	object
			16	Apparent Cause of Accident	object
			17	Deaths happens	object
			18	Injury happens	object

**Table 2:** Experimental scenarios used in the APG-GRU framework.

Scenario	Feature/Model Combination	Purpose
Scenario 1	ANPR Traffic Data + Peak-Hour Features + Fuzzy Labeling + GRU	Baseline temporal traffic prediction using vehicle flow and fuzzy congestion features
Scenario 2	ANPR Traffic Data + Peak-Hour Features + Fuzzy Labeling + GAN Balancing + GRU	Test effect of GAN balancing without accident features
Scenario 3	ANPR Traffic Data + Accident Features + Peak-Hour Features + Fuzzy Labeling + GRU	Test effect of accident features without GAN balancing
Scenario 4	ANPR Traffic Data + Accident Features + Peak-Hour Features + Fuzzy Labeling + GAN Balancing + GRU (Proposed APG-GRU Framework)	Complete proposed framework combining recurring and non-recurring features

**Table 3:** Evaluation metrics used for scenario comparison.

Metric	Formula
Accuracy	(TC + TS)/(TC + TS + FC + FS)
Congested Precision	TC/(TC + FC)
Congested Recall	TC/(TC + FS)
Congested F1-score	2 × (Congested Precision × Congested Recall)/(Congested Precision + Congested Recall)

**Table 4:** Scenario-wise performance comparison of the proposed APG-GRU framework on the Islamabad dataset.

Scenario	Feature/Model Combination	Accuracy (%)	Congested Precision (%)	Congested Recall (%)	Congested F1-Score (%)
Scenario 1	ANPR Traffic Features + Peak-Hour Features + Fuzzy Labeling + GRU	86.50	64.49	86.64	73.94
Scenario 2	ANPR Traffic Features + Peak-Hour Features + Fuzzy Labeling + GAN Balancing + GRU	90.50	73.02	90.45	80.80
Scenario 3	ANPR Traffic Features + Accident Features + Peak-Hour Features + Fuzzy Labeling + GRU	94.50	82.91	94.62	88.38
Scenario 4	ANPR Traffic Features + Accident Features + Peak-Hour Features + Fuzzy Labeling + GAN Balancing + GRU (APG-GRU framework)	97.50	91.86	97.31	94.51

**Table 5:** Comparative performance of Random Forest, XGBoost, and LSTM across the four experimental scenarios on the Islamabad dataset.

Model	Scenario	Accuracy (%)	Congested Precision (%)	Congested Recall (%)	Congested F1-Score (%)
Random Forest	Scenario 1	93.7	88.7	82.0	85.2
Random Forest	Scenario 2	93.8	88.7	82.7	85.6
Random Forest	Scenario 3	96.3	93.2	89.9	91.5
Random Forest	Scenario 4	96.3	93.3	89.7	91.5
XGBoost	Scenario 1	93.1	78.8	94.3	85.8
XGBoost	Scenario 2	93.1	78.8	94.2	85.8
XGBoost	Scenario 3	96.0	86.9	96.2	91.3
XGBoost	Scenario 4	95.9	86.6	96.3	91.2
LSTM	Scenario 1	92.8	77.8	94.3	85.3
LSTM	Scenario 2	92.8	77.4	95.0	85.3
LSTM	Scenario 3	95.8	86.0	96.7	91.0
LSTM	Scenario 4	95.5	84.9	96.8	90.5

**Table 6:** Repeated-run statistical comparison of APG-GRU with baseline models.

Model	Accuracy Mean ± SD	95% CI	Congested Precision Mean ± SD	95% CI	Congested Recall Mean ± SD	95% CI	Congested F1-Score Mean ± SD	95% CI
Random Forest	93.345 ± 0.538	[92.677, 94.013]	90.284 ± 1.202	[88.791, 91.776]	82.559 ± 0.911	[81.428, 83.689]	86.241 ± 0.565	[85.540, 86.943]
XGBoost	94.395 ± 0.401	[93.898, 94.893]	86.944 ± 1.049	[85.641, 88.246]	91.574 ± 1.103	[90.206, 92.943]	89.190 ± 0.416	[88.674, 89.706]
LSTM	81.615 ± 1.398	[79.879, 83.351]	59.586 ± 3.098	[55.740, 63.432]	85.215 ± 3.694	[80.628, 89.801]	70.042 ± 1.944	[67.628, 72.455]
GRU	86.102 ± 1.129	[84.701, 87.504]	67.813 ± 2.010	[65.318, 70.308]	85.472 ± 2.093	[82.873, 88.071]	75.620 ± 1.957	[73.190, 78.049]
STGCN	86.659 ± 1.067	[85.335, 87.984]	67.758 ± 1.151	[66.329, 69.186]	90.089 ± 1.540	[88.176, 92.001]	77.333 ± 0.872	[76.251, 78.416]
DCRNN	86.757 ± 1.061	[85.440, 88.074]	67.973 ± 1.119	[66.584, 69.362]	90.033 ± 1.651	[87.983, 92.083]	77.452 ± 0.828	[76.423, 78.480]
APG-GRU	97.375 ± 0.265	[97.045, 97.704]	92.377 ± 0.901	[91.258, 93.496]	97.590 ± 0.811	[96.583, 98.598]	94.911 ± 0.799	[93.919, 95.903]

**Table 7:** Scenario-wise performance comparison of the proposed APG-GRU framework on the Lahore dataset.

Scenario	Feature/Model Combination	Accuracy (%)	Congested Precision (%)	Congested Recall (%)	Congested F1-Score (%)
Scenario 1	ANPR Traffic Features + Peak-Hour Features + Fuzzy Labeling + GRU	91.78	74.30	96.17	83.83
Scenario 2	ANPR Traffic Features + Peak-Hour Features + Fuzzy Labeling + GAN Balancing + GRU	92.23	75.93	95.04	84.42
Scenario 3	ANPR Traffic Features + Accident Features + Peak-Hour Features + Fuzzy Labeling + GRU	95.29	84.09	97.13	90.14
Scenario 4	ANPR Traffic Features + Accident Features + Peak-Hour Features + Fuzzy Labeling + GAN Balancing + GRU (APG-GRU framework)	95.41	84.74	96.69	90.32

**Table 8:** Comparative performance of Random Forest, XGBoost, and LSTM across the four experimental scenarios on the Lahore dataset.

Model	Scenario	Accuracy (%)	Congested Precision (%)	Congested Recall (%)	Congested F1-Score (%)
Random Forest	Scenario 1	94.25	89.50	83.82	86.57
Random Forest	Scenario 2	94.27	89.44	83.99	86.63
Random Forest	Scenario 3	94.42	92.99	90.65	91.81
Random Forest	Scenario 4	94.35	92.85	90.43	91.63
XGBoost	Scenario 1	92.91	77.99	94.73	85.55
XGBoost	Scenario 2	92.91	78.11	94.47	85.52
XGBoost	Scenario 3	94.67	86.29	95.65	90.73
XGBoost	Scenario 4	94.72	86.38	95.78	90.83
LSTM	Scenario 1	92.82	78.12	93.86	85.27
LSTM	Scenario 2	91.73	74.10	96.30	83.76
LSTM	Scenario 3	94.27	83.92	96.26	90.10
LSTM	Scenario 4	94.07	86.09	96.47	90.99

## Data Availability

Data available on request from the authors.
